# Comparative transcriptome analyses of the *Drosophila* pupal eye

**DOI:** 10.1093/g3journal/jkaa003

**Published:** 2020-11-20

**Authors:** Miles W DeAngelis, Joseph D Coolon, Ruth I Johnson

**Affiliations:** Department of Biology, Wesleyan University, 52 Lawn Avenue, Middletown, CT 06459, USA

**Keywords:** pupae, eye, *Drosophila*, transcriptome, morphogenesis

## Abstract

Tissue function is dependent on correct cellular organization and behavior. As a result, the identification and study of genes that contribute to tissue morphogenesis is of paramount importance to the fields of cell and developmental biology. Many of the genes required for tissue patterning and organization are highly conserved between phyla. This has led to the emergence of several model organisms and developmental systems that are used to study tissue morphogenesis. One such model is the *Drosophila melanogaster* pupal eye that has a highly stereotyped arrangement of cells. In addition, the pupal eye is postmitotic that allows for the study of tissue morphogenesis independent from any effects of proliferation. While the changes in cell morphology and organization that occur throughout pupal eye development are well documented, less is known about the corresponding transcriptional changes that choreograph these processes. To identify these transcriptional changes, we dissected wild-type *Canton S* pupal eyes and performed RNA-sequencing. Our analyses identified differential expression of many loci that are documented regulators of pupal eye morphogenesis and contribute to multiple biological processes including signaling, axon projection, adhesion, and cell survival. We also identified differential expression of genes not previously implicated in pupal eye morphogenesis such as components of the Toll pathway, several non-classical cadherins, and components of the muscle sarcomere, which could suggest these loci function as novel patterning factors.

## Introduction

**Figure 1:**
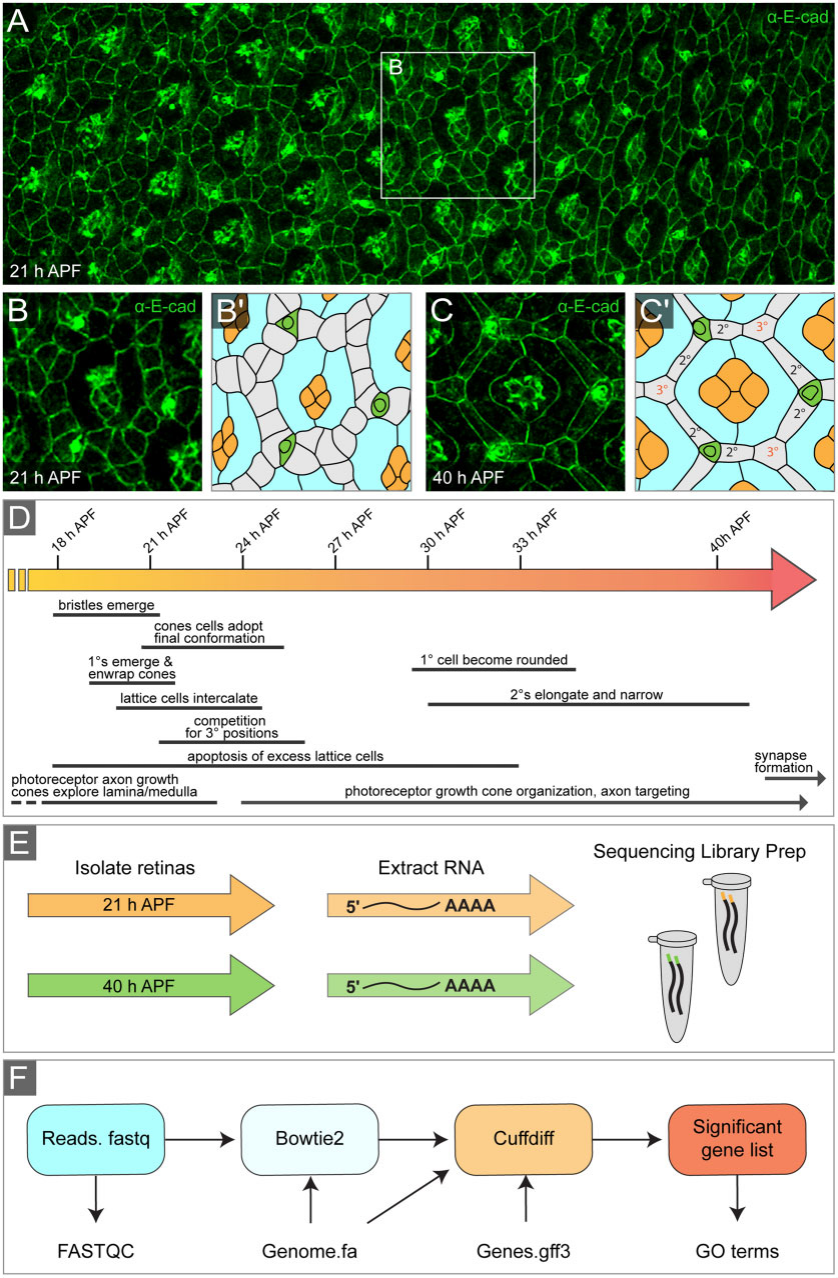
The *Drosophila* pupal eye and experimental design. (A) Mid-region of a pupal eye at 21 h APF, characterized by a developmental gradient. Younger, anterior (less organized) tissue to the left and older (posterior) tissue is to the right. White box encloses a single ommatidium shown at higher resolution in (B) with tracing (B′). (C) Representative ommatidium at 40 h APF and tracing (C′), with cone cells colored orange, 1° cells in blue, bristle groups in green, and lattice cells in gray with 2° and 3° cells indicated. (D) Timeline of development of the pupal eye. Major morphogenetic events that occur between 18 and 40 h APF are indicated. Times relate to development at 25°C. (E) Experimental workflow: eyes were dissected at 21 or 40 h APF, total RNA extracted, and sequencing libraries prepared (cDNA synthesis and bar-coding). (F) Bioinformatics pipeline used to identify differentially expressed genes and identify enriched gene ontology terms from raw sequencing reads.

The *Drosophila* pupal eye is a postmitotic pseudostratified neuroepithelium that is organized into ∼750 optical units known as ommatidia (Figure 1, A–D). Each ommatidium contains eight photoreceptor neurons (R1–R8), four lens-secreting cone cells, and two pigment-producing primary (1°) cells (Ready *et al.* 1976; Cagan and Ready 1989a; Wolff and Ready 1991a, 1993; Carthew 2007). Surrounding each ommatidium are lattice cells, which also produce pigment. By 40 h after puparium formation (APF), the pupal eye has achieved its stereotypical honeycomb organization and lattice cells can be classified as either secondary (2°) or tertiary (3°) cells depending on the number of contacts they form with adjacent ommatidia. Those classified as 2° cells have an elongated rectangular apical surface area that contacts two adjacent ommatidia, while the apical surface area of 3° cells is more hexagonal in shape and contacts three adjacent ommatidia (Figure 1C). In addition, each eye contains ∼600 sensory bristle groups that are present at the anterior vertex of each ommatidium with the exception of those along the edges of the eye (Wigglesworth 1953; Waddington and Perry 1960; Cagan and Ready 1989a).

During embryogenesis, the first cells are selected that will go on to form the eyes (Honn *et al.* 2016). These retinal progenitor cells proliferate to form the eye-antennal imaginal disks that give rise to the eyes as well as the antennae, ocelli, and surrounding head epithelium (Haynie and Bryant 1986). Establishment of the eye disk and the subsequent eye field is dependent on a network of highly conserved transcription factors known as the Retinal Determination Network (Kumar 2010, 2011; Treisman 2013). The major components of this network include Eyeless, Optix, Dachshund, Twin of eyeless, Eyegone, Eyes absent, and Sine oculis (Cheyette *et al.* 1994; Mardon *et al.* 1994; Quiring *et al.* 1994; Serikaku and O'Tousa, 1994; Halder *et al.* 1995; Czerny *et al.* 1999; Seimiya and Gehring 2000; Jang *et al.* 2003). At the beginning of the third larval instar, a combination of Hedgehog (Hh) (Wolff and Ready 1991a; Ma *et al.* 1993; Ma and Moses 1995; Treisman and Rubin 1995; Greenwood and Struhl 1999; Curtiss and Mlodzik 2000; Kenyon *et al.* 2003) and Ecdysone (Bate and Martínez-Arias 1993; Thummel 1996; Niwa *et al.* 2004) signaling leads to the initiation and progression of the morphogenetic furrow. The furrow, which is caused by ingression of the apical surface area of columns of retinal progenitor cells, is initiated from the posterior of the eye disk and progresses toward the anterior over the course of several hours. This progression leads to the formation a developmental gradient that persists throughout most of eye morphogenesis (Wolff and Ready 1991a).

Cell differentiation in the eye is stepwise, beginning during passage of the morphogenetic furrow with the specification of the eight photoreceptors (Reinke and Zipursky 1988; Van Vactor *et al.* 1991; Jarman *et al.* 1994, 1995; Fanto and Mlodzik 1999; Frankfort *et al.* 2001; Pepple *et al.* 2008). Shortly after their recruitment, photoreceptors begin to project their axons to the lamina or the medulla (Gibbs and Truman 1998; Clandinin and Zipursky 2002; Pepple *et al.* 2008). Axons will reach their target brain regions at ∼24 h APF and begin to form synapses with target neurons at ∼50 h APF (Gibbs and Truman 1998). Over the course of pupal development, photoreceptors undergo substantial morphological changes. Beginning at ∼48 h APF, the apical poles of each photoreceptor fold 90° toward one another. The central region of this apical pole will develop into the rhabdomere, which contains rhodopsin-rich microvilli (Knust 2007). Recruitment of cone and 1° cells, as well as bristle groups, occurs after photoreceptor specification. Photoreceptors initiate cone cell recruitment through a combination of epidermal growth factor receptor (EGFR) and Notch signaling (Tomlinson and Ready 1987; Cagan and Ready 1989b; Zak and Shilo 1992; Freeman 1996, 1997). Over the course of pupal eye development cone cell contacts become highly stereotyped, facilitated by the expression of N-cadherin (Hayashi and Carthew 2004). After cone cell specification, EGF secreted by photoreceptors initiates the expression of the Notch ligand Delta on the surface of cone cells (Nagaraj and Banerjee 2007). The interaction between Delta and the Notch receptor, expressed on the surface of retinal progenitors, leads to the specification of two cells that become 1° cells (Cagan and Ready 1989b; Nagaraj and Banerjee 2007). Once selected, the 1° cells gradually enwrap the four cone cells. Formation of the bristle groups occurs at ∼18 h APF when two cells are selected to undergo a final round of mitosis, giving rise to the four cells that comprise each group (Cagan and Ready 1989a; Meserve and Duronio 2017). This will be the final mitotic division to take place during *Drosophila* eye development with all remaining retinal progenitor cells adopting the lattice cell fate.

Pupal eye development is dependent on the precise regulation of adhesion between cells. In the eye, adhesion is directed by E-cadherin (Grzeschik and Knust 2005; Larson *et al.* 2008; Seppa *et al.* 2008; Zaessinger *et al.* 2015) and the immunoglobulin (Ig) domain adhesion molecules Roughest (Rst), Kin of Irre (Kirre), Hibris (Hbs), and Sticks and Stones (Sns) (Gorski *et al.* 2000; Araujo *et al.* 2003; Bao and Cagan 2005; Grzeschik and Knust 2005; Mirkovic and Mlodzik 2006; Grillo‐Hill and Wolff 2009; Bao *et al.* 2010). The expression and interaction of Ig domain adhesion molecules is complimentary. Rst and Kirre are expressed in lattice cells, while Hbs and Sns are expressed in 1° cells (Gorski *et al.* 2000; Bao and Cagan 2005; Bao *et al.* 2010). In the eye, Rst interacts with Hbs, while Sns interacts with Kirre and these interactions are required for cell sorting and maintaining preferential adhesion between 1° and lattice cells within the developing eye (Bao and Cagan 2005; Grillo‐Hill and Wolff 2009; Bao *et al.* 2010). Precise regulation of adhesion is also required for intercalation that organizes lattice cells into a single row surrounding each ommatidium. Intercalation occurs between ∼18 and 27 h APF and is dependent on interactions between Rst and Hbs, as well as interactions between Cindr and the ArfGAPs that regulate Arf6 (Johnson *et al.* 2008, 2011; Larson *et al.* 2008).

Each pupal eye is equipped with more lattice cells than needed for eventual formation of the honeycomb lattice leading to the apoptosis of approximately one-third of lattice cells between ∼18 and 33 h APF. Lattice cell removal is directed by a combination of Wingless, JNK, and Notch signaling (Cagan and Ready 1989b; Wolff and Ready 1991b; Cordero *et al.* 2004; Bushnell *et al.* 2018). Survival of individual lattice cells is dependent on EGFR and Yorkie activities, a lattice cell’s proximity to 1° cells, and the balance of death and survival signals that a cell receives (Rusconi *et al.* 2000; Monserrate and Brachmann 2007; DeAngelis *et al.* 2020).

While the morphological events that occur during pupal eye development have been well documented, little is known about the corresponding transcriptional changes that facilitate and choreograph them. Previous transcriptome studies of *Drosophila* eyes have focused on the larval eye disk (Ikmi *et al.* 2014; Potier *et al.* 2014; Torres-Oliva *et al.* 2018), adult eye (Hall *et al.* 2017), or analyzed transcriptional changes throughout the entire pupal head (Ranade *et al.* 2008). In this study, we compare the transcriptomes of pupal eyes at 21 and 40 h APF using RNA-sequencing (RNA-seq) to capture differences in the expression of genes associated with adhesion, cell death, axon projection, and the signaling pathways that regulate these developmental processes. Our analysis identified large-scale transcriptional differences between the two developmental ages. Some of the differentially expressed genes we identified have previously been established as regulators of pupal eye morphogenesis, or have been implicated in signal transduction, axon projection, adhesion, or cell survival in the eye or other tissues. In addition, we identify many novel genes not yet associated with eye development. These included members of the Toll signaling pathway, several non-classical cadherins, and genes associated with muscle structure and development. We anticipate that the transcriptome data presented here will be a valuable resource for *Drosophila* pupal eye biologists and the broader morphogenesis field.

## Materials and methods

### Fly stocks

*Drosophila melanogaster Canton S* cultures were maintained at 25°C on nutrient-rich medium.

### Immunofluorescence

Pupal eyes were dissected and fixed as previously described (DeAngelis and Johnson 2019). For 1° antibody staining, rat anti-E-cad (1:20, DSHB, # 528120) was used to visualize cell boundaries. Secondary antibodies conjugated to Alexa Fluor^®^ 488 (Jackson ImmunoResearch) were used at 1:200. Dissected pupal eyes were imaged with a Leica DM5500 B fluorescence microscope and corresponding software.

### RNA-seq and bioinformatics analysis

**Figure 2:**
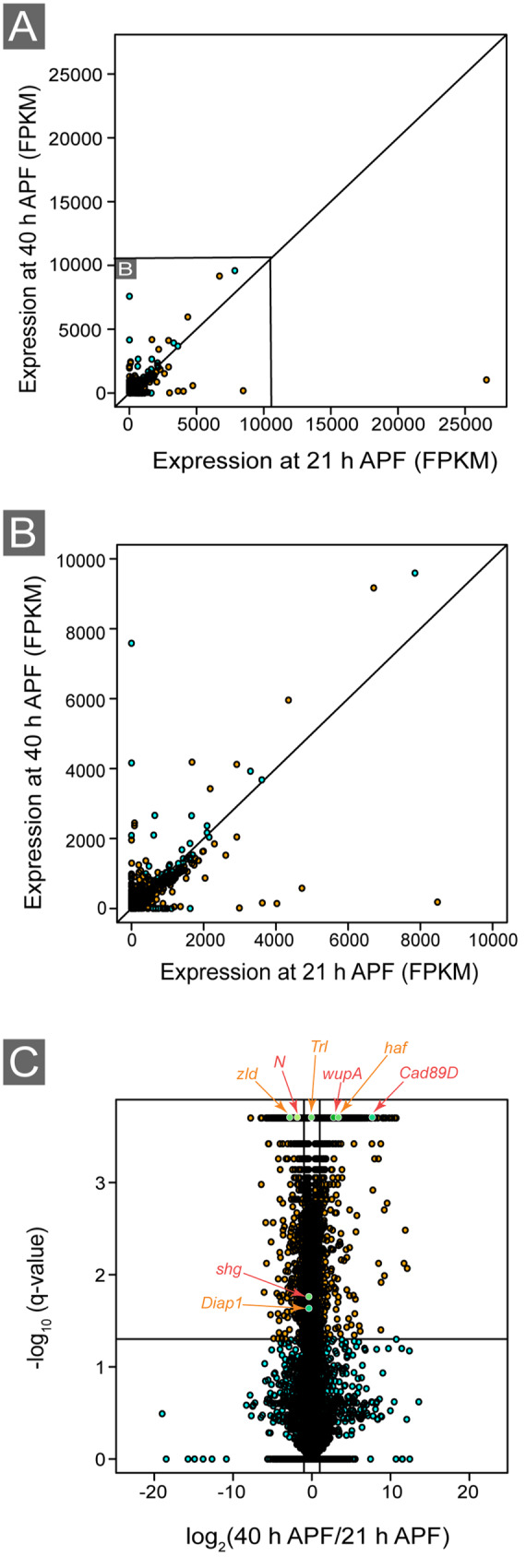
Graphical representation of gene expression data. (A) Scatterplot of expression fragments per kilobase of transcript per million mapped reads of loci at 21 h APF (*x*-axis) and 40 h APF (*y*-axis). (B) Higher-resolution plot of boxed region in (A). (C) Volcano plot of gene expression difference (*x*-axis) against −log_10_-transformed statistical significance (*q*-values). Horizontal line represents *q*-value = 0.05. (A–C) Statistically significant differentially expressed genes are indicated in orange (*q*-value <0.05) and several of these genes that are discussed in the text are indicated in green. Non-statistically significant genes are indicated in cyan (*q*-value >0.05).

Between 50 and 70 eyes were dissected from 21 and 40 h APF *Canton S* pupae at the same time each day, and total RNA was extracted from three biological replicates using the ReliaPrep RNA Tissue Miniprep System (Promega Corporation, Cat # M3001) as described (DeAngelis and Johnson 2019). Barcoded cDNA library prep was performed using the TruSeq library preparation kit, libraries were pooled, balanced pooling was confirmed using qPCR, and 51-bp paired-end sequencing was performed by the University of Michigan Sequencing Core Facility as described (DeAngelis *et al.* 2020). All raw sequencing reads were imported into Galaxy (https://usegalaxy.org/). Quality control of sequence read outputs was performed using FASTQC (Andrews, 2010; Afgan *et al.* 2018). The percentage of mapped reads was calculated using FlagStat (Li *et al.* 2009). Sequence reads were aligned to the *D. melanogaster* reference genome available from Ensembl (Zerbino *et al.* 2018) at the time of analysis: reference genome: Drosophila_melanogaster.BDGP6.dna.toplevel.fa and gene annotation: Drosophila_melanogaster.BDGP6.93.gff3 (Zerbino *et al.* 2018) using bowtie2 with default parameters (Langmead and Salzberg 2012). Gene expression quantification along with corresponding statistical analyses were performed using Cuffdiff. Cuffdiff parameters included geometric normalization and transcript length correction where a bias length correction using the reference genome was performed (Trapnell *et al.* 2012). Significantly differentially expressed genes were identified as those with false discovery rate corrected *P*-values (*q*-values) lower than our predetermined threshold (*q* < 0.05). Gene ontology (GO) analyses were performed using the Gene Ontology Consortium (http://geneontology.org/). Volcano and scatter plots were created with R-statistical software (Figure 2) (CRAN 2018).

Candidate regulatory transcription factors and transcription factor-binding motifs were identified with i-Cis Target analysis (https://gbiomed.kuleuven.be/apps/lcb/i-cisTarget/index.php) (Herrmann *et al.* 2012) and Analysis of Motif Enrichment (AME) (http://meme-suite.org/tools/ame) (McLeay and Bailey 2010) using lists of candidate genes and default parameters. Prior to AME analyses, the extended gene region for each significantly differentially expressed gene was downloaded from FlyBase (https://flybase.org/) using batch download (https://flybase.org/download/sequence/batch/).

### Data availability statement

Raw RNA-seq output files generated in this work are deposited under accession number GSE160441 in Gene Expression Omnibus.

Supplementary material is available at figshare DOI: https://doi.org/10.25387/g3.12824789.

## Results and discussion

### Mapped reads and GO

**Figure 3:**
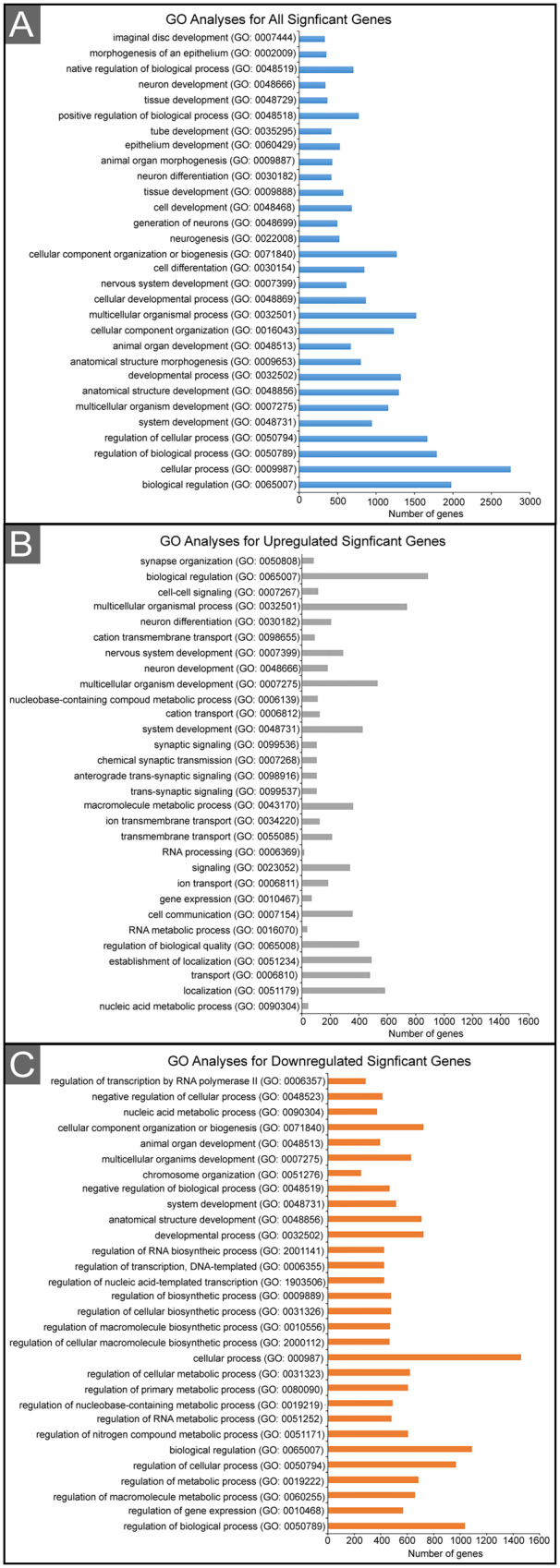
Graphical representation of significant GO terms. The 30 most-significant GO terms (A) when comparing all significant gene expression changes from 21 and 40 h APF, (B) loci with increased expression at 40 h relative to 21 h APF, (C) loci with decreased expression at 40 h relative to 21 h APF.

To identify transcriptional changes during pupal eye morphogenesis, we dissected *Canton S* eyes at 21 and 40 h APF (Figure 1, A–C) (Materials and methods). We chose to analyze differences in gene expression at 21 h APF as the eye is in the midst of critical patterning and developmental events (Figure 1D). These include 1° cell recruitment, lattice cell intercalation, the conclusion of axon outgrowth into the brain, and the beginning of elimination of excess lattice cells by apoptosis. We selected 40 h APF for a comparison point as apoptosis has ceased, the honeycomb lattice is established, and axon growth cones are approaching their future synaptic targets. After dissection, total RNA was extracted and sequenced (Figure 1E) (Materials and methods). A total of 2.7 × 10^8^ sequence reads were generated with a range of 40,214,252–44,584,127 mapped reads per sample (92.65–93.42% mapped to the reference genome) indicating appropriate read depth for analysis (Table 1). Sequence reads were then subjected to an established bioinformatics pipeline to identify differentially expressed genes (Figure 1F) (Materials and methods) (Lanno *et al.* 2017). Our analyses identified 4636 loci in which expression was significantly modified between 21 and 40 h APF (*q*-value <0.05) (Figure 2 and Supplementary Table S1). GO terms, identified by the Gene Ontology Consortium with the lowest *P*-values for all differentially expressed genes between 21 and 40 h APF included biological regulation (GO:0065007), cellular process (GO:0009987), regulation of biological process (GO:0050789), and regulation of cellular process (GO:0050794) (Figure 3A and Supplementary Table S2). Of the 4636 genes, we identified the increased expression of 2383 genes at 40 h relative to 21 h APF and decreased expression of 2253 genes demonstrating no bias in the direction of regulation (binomial exact test, *P *=* *0.05813). The GO terms that were most significant for upregulated genes included nucleic acid metabolic process (GO:0090304), localization (GO:0051179), transport (GO:0006810), and establishment of localization (GO:0051234) (Figure 3B and Supplementary Table S3). For downregulated genes, the most significant GO terms included regulation of biological process (GO:0050789), regulation of gene expression (GO:0010468), macromolecule metabolic process (GO:0060255), and regulation of metabolic process (GO:0019222) (Figure 3C and Supplementary Table S4).

**Table 1:** Total number of reads and mapped reads per sequencing library This table has not been correctly formatted when converted from the file submitted. There are two Rep 1 entries under the heading 21 h APF. The second should be under the heading 40 h APF instead.

	Total reads	Mapped reads	% mapped
21 h APF			
Rep1	44,711,158	41,768,811	93.42
Rep2	43,337,942	40,376,753	93.17
Rep3	43,677,528	40,768,017	93.34
Rep1 (this should be underthe 40 h APF	48,119,480	44,584,127	92.65
40 h APF			
Rep2	46,431,166	43,315,962	93.29
Rep3	43,313,444	40,214,252	92.84

### Differential expression of signaling pathway components between 21 and 40 h APF

Since development of the pupal eye is dependent on signal transduction, we assessed whether expression of core signaling proteins differed at 21 and 40 h APF. Our analyses identified differential expression of multiple loci broadly associated with signaling (GO:0023052) (Supplementary Table S5). These loci encompassed core components of several signaling pathways with established roles during *Drosophila* pupal eye development including the Notch, EGFR, Decapentaplegic (Dpp)/TGFβ, and Planar Cell Polarity pathways and also identified signaling pathways that have not been interrogated for their role in fly eye development (Supplementary Table S5).

Notch signaling is vital during early pupal eye development for photoreceptor, cone and 1° cell specification, patterning, and inducing cell death (Cagan and Ready 1989b; Fortini *et al.* 1993; Cordero *et al.* 2004; Grzeschik and Knust 2005; Nagaraj and Banerjee 2007; Bao *et al.* 2010; Nagel and Preiss 2011; Xiong *et al.* 2013; Bao 2014). At 40 h relative to 21 h APF, we observed a reduction in the expression of the Notch receptor (*N*) and its ligand *Delta* (*Dl*) (Figure 2C) (Vässin and Campos-Ortega 1987; Rebay *et al.* 1991; Fleming *et al.* 1997) but an increase in the ligands *Serrate* (Rebay *et al.* 1991) and *weary* (*wry*) (Kim *et al.* 2010). Expression of the Notch regulators *O-fucosyltransferase 1* (*O-fut1*) (Sasamura *et al.* 2003) and *Notchless* (*Nle*) (Royet *et al.* 1998) decreased by 40 h APF, as did *mind bomb 1* (*mib1*) (Lai *et al.* 2005) and the DNA-binding transcription factor *Suppressor of Hairless* (*Su(H)*) (Gho *et al.* 1996) that are also required for N signaling. Consistent with reduced Notch pathway activity at 40 h APF, we observed a significant reduction in the expression of 10 *enhancers of split* (*E(spl)*) genes that are transcriptional targets of Notch signaling (Schrons *et al.* 1992; Jennings *et al.* 1994) (Supplementary Table S1).

The EGFR pathway is required for cell differentiation, adhesion, and survival during early pupal eye development (Wasserman *et al.* 2000; Freeman and Bienz 2001; Brown and Freeman 2003; Protzer *et al.* 2008; Martín-Bermudo *et al.* 2015; Malartre 2016). We therefore reasoned that, similar to components of the Notch pathway, we would observe reduced expression of EGFR pathway components at 40 h relative to 21 h APF as differentiation, apoptosis, and patterning have concluded. Consistent with this prediction, we detected significant reduction in the expression of several canonical Receptor Tyrosine Kinase (RTK) components including *Egfr* (Schejter and Shilo 1989), the adaptor proteins *downstream of receptor kinase* (*drk*), and *SHC adaptor protein* (*Shc*) (Stern *et al.* 1993; Lai *et al.* 1995), the GEF *Son of sevenless* (*Sos*) (Rogge *et al.* 1991), the small GTPase *Ras oncogene at 85D* (*Ras85D*) (Neuman-Silberberg *et al.* 1984), the kinases *Raf oncogene* (*Raf*) (Mark *et al.* 1987) and *Downstream of raf1* (*Dsor1*) (Lu *et al.* 1993), and the transcriptional activator *pointed* (*pnt*) (Scholz *et al.* 1993) (Supplementary Table S5). While the expression of *Egfr* and many core RTK signaling components declined at 40 h APF, expression of several other RTKs and their ligands increased (Supplementary Table S5) suggestive of a continuous requirement for RTK-components in the eye. These included *PDFG and VEGF receptor related* (*Pvr*) (Heino *et al.* 2001) along with its ligands *PDFG and VEGF receptor-related factor 1* (*Pvf1*) (Duchek *et al.* 2001), *Pvf2* (Munier *et al.* 2002), *Pvf3* (Cho *et al.* 2002), and both *branchless* (*bnl*) (Sutherland *et al.* 1996) and *heartless* (*htl*) (Beiman *et al.* 1996), which encode a fibroblast growth factor and fibroblast growth factor receptor (FGFR), respectively. Since FGFR signaling regulates E-cadherin, crumbs, and actin expression in the developing *Drosophila* pupal eye, increased expression of *bnl* and *htl* could contribute to the maintenance of adhesion during later developmental stages (Mukherjee *et al.* 2012). In addition, these results could be suggestive of a prominent role for RTK’s such as *pvr* to mediate cell survival during the later stages of pupal eye development as is observed in blood and neural progenitor cells (Brückner *et al.* 2004; Read 2018).

Our data also revealed differential expression of signaling proteins, which have not yet been implicated as regulators of pupal eye development. For example, we found that core components of the Toll signaling pathway (Supplementary Table S5) were differentially expressed between 21 and 40 h APF including the secreted protease *Spatzle-Processing Enzyme* (*SPE*) (Jang *et al.* 2006), the spatzle cleaving enzyme *easter* (*ea*) (Chasan and Anderson 1989), the receptors *Toll* (*Tl*) (Anderson *et al.* 1985), *Tollo* (Seppo *et al.* 1999), *Toll-6*, *Toll-7* (Tauszig *et al.* 2000), and *18 wheeler* (*18w*) (Williams *et al.* 1997), the scaffolding protein *Myd88* (Horng and Medzhitov 2001), and the transcription factor *dorsal* (*dl*) (Lemaitre *et al.* 1995). The Toll pathway has been widely studied in the context of the immune response (Valanne *et al.* 2011; Satoh and Akira 2017; Vidya *et al.* 2018); however, it was first identified as a regulator of dorsal ventral patterning of the embryo (Anderson *et al.* 1985; Morisato and Anderson 1994). Toll signaling has also been implicated during wound healing of the *Drosophila* embryonic epidermis (Carvalho *et al.* 2014; Capilla *et al.* 2017) and for epithelial integrity in the *Drosophila* salivary gland (Kolesnikov and Beckendorf 2007). In addition, the Toll receptors Toll-2 (encoded by *18w*), Toll-6 (encoded by *Toll-6*) and Toll-8 (encoded by *tollo*) form transheterophillic complexes that facilitate cell intercalation in the embryonic epithelium by stimulating actin reorganization (Paré *et al.* 2014). Expression of each of these three toll receptors increased at 40 h APF relative to 21 h APF (Supplementary Table S5) and studies that assess their role during pupal eye morphogenesis are an interesting avenue of future research.

### Differential expression of Ecdysone pathway components and response elements

Ecdysone is a steroid hormone required for insect metamorphosis that facilitates the transition from the larval to pupal stage (Becker 1959; Ashburner *et al.* 1974; Bate and Martínez-Arias 1993; Thummel 1996). During pupal development, ecdysone levels peak at ∼20 h APF and progressively drop until adults eclose (Thummel 2001). In addition, our analyses identified increased expression of *abrupt* (*ab*) at 40 h relative to 21 h APF, which negatively regulates the transcription of ecdysone target genes (Jang *et al.* 2009) (Supplementary Table S1). We therefore reasoned that expression of documented Ecdysone transcriptional target genes would be reduced at 40 h APF and this was indeed the case for *Edg91* (Apple and Fristrom 1991), *Eip71CD* (Savakis *et al.* 1980), *Eip63E* (Stowers *et al.* 2000), *ftz-f1* (Woodard *et al.* 1994), *ImpE2* and *ImpL2* (Natzle *et al.* 1988), and *Pep* (Möritz *et al.* 1984) (Supplementary Table S6). However, the expression of other ecdysone response element genes increased, including *Eip93F* (Baehrecke and Thummel 1995), *Eip74EF* (Burtis *et al.* 1990), *Eip78C* (Stone and Thummel 1993), and *Eip75B* (Segraves and Hogness 1984) (Supplementary Table S6). Since ecdysone signaling contributes to apoptosis in other *Drosophila* pupal tissues including the salivary gland, midgut, and muscle (Denton and Kumar 2015; Nicolson *et al.* 2015; Xu *et al.* 2019), it is possible that ecdysone response genes identified in our analyses of 21 h APF eyes contribute to or even initiate the apoptosis of lattice cells, which begins at around 18–20 h APF, correlating with increased ecdysone (Thummel 2001).

### Gene expression changes associated with axon projection

Growth of photoreceptor axons begins during larval development and continues until axons reach the lamina or medulla at ∼24 h APF (Gibbs and Truman 1998). Axons then extend exploratory growth cones (from ∼27 h APF) so that synaptogenesis can begin from ∼50 h APF (Gibbs and Truman 1998; Clandinin and Zipursky 2002). Accordingly, we identified differential expression of loci associated with axon elongation and extension, growth cone formation, synaptogenesis, and neuronal signaling (Supplementary Table S7). These included *hattifattener* (*haf*), which increased in expression at 40 h relative to 21 h APF (Supplementary Table S7 and Figure 2C). The *haf* locus encodes a protein required for axon targeting and increased expression is indicative of a maturing neural system (Kurusu *et al.* 2008). Expression of several cell adhesion molecules also increased at 40 h APF. These included the neural-cadherins *CadN* (Iwai *et al.* 1997) and *CadN2* (Yonekura *et al.* 2007), *Turtle* (*Tutl*) (Al-Anzi and Wyman 2009), and 10 different BEAT-family genes (Pipes *et al.* 2001). Collectively, these adhesion molecules are required for axon projection and synapse formation (Longley and Ready 1995; Iwai *et al.* 1997; Knust 2007) and increased expression of these loci is consistent with axon extension.

Growth cone extension is an integral part of axon projection and is dependent on dynamic cytoskeletal rearrangements. Accordingly, we found differential expression of genes known to be required for actin remodeling at 40 h relative to 21 h APF consistent with the actin architectural rearrangements necessary for growth cone extension (Supplementary Table S7). For example, we identified increased transcription of *Capulet* (*Capt*), which inhibits actin filament growth (Wills *et al.* 2002), *enabled* (*ena*), which stimulates the addition of actin monomers and is required for axon elongation (Wills *et al.* 1999; Bashaw *et al.* 2000), and *cherrio* (*cher*), a filament protein that crosslinks actin filaments (Sokol and Cooley 1999). We found decreased expression of *chickadee* (*chic*), which encodes profilin (Cooley *et al.* 1992), *jitterbug* (*jbug*), which encodes an actin cross-linking protein (Oliva *et al.* 2015), and *cdc42*, which activates the WASP complex (Luo *et al.* 1994). In addition to the differential expression of loci associated with actin regulation, we identified increased expression of loci associated with microtubules that promote axon elongation and maturation. These included *Tau* (Bolkan and Kretzschmar 2014), which crosslinks microtubules in the axon, and *Kinesin-like protein at 64D* (*Klp64D*), a motor protein associated with axonal transport (Berger *et al.* 2008). Taken together, these data are consistent with the dynamic cytoskeletal rearrangements consistent with growth cone extension.

### Differential expression in cell adhesion regulators during pupal eye development

Modulations in adhesion are vital as cells undergo complex shape and positional changes during morphogenesis (Collinet and Lecuit 2013; Guillot and Lecuit 2013; Martin and Goldstein 2014; Takeichi 2014; McFaul and Fernandez-Gonzalez 2017; Pannekoek *et al.* 2019). Accordingly, we found significant changes in the expression of many cell adhesion loci (Supplementary Table S8). For example, at 40 h APF, we detected decreased expression of the Ig domain adhesion molecules *rst* (Ramos *et al.* 1993), *hbs* (Dworak *et al.* 2001), and *sns* (Bour *et al.* 2000), which are required for pigment cell morphogenesis (Araujo *et al.* 2003; Bao and Cagan 2005; Grzeschik and Knust 2005; Grillo‐Hill and Wolff 2009; Bao *et al.* 2010) and photoreceptor axon guidance (Schneider *et al.* 1995; Sugie *et al.* 2010), although we did not identify a significant change in the expression of the Sns ligand *Kirre* (Strünkelnberg *et al.* 2001) (log_2_-fold change = 0.05). Since Notch signaling regulates the expression of *hbs* (Bao 2014), the reduced Notch activity at 40 h APF may explain the reduction in its expression (Supplementary Tables S1, S5, and S8). We also identified decreased expression of *shg* (encodes E-cad) by 40 h APF (Figure 2C) (Tepass *et al.* 1996), which is required for adhesion in the developing pupal eye (Grzeschik and Knust 2005; Larson *et al.* 2008; Seppa *et al.* 2008; Zaessinger *et al.* 2015). Taken together, reduced expression of these adhesion molecules at 40 h APF may reflect a lower requirement once the honeycomb lattice is established.

Our analyses also highlighted changes in the expression of many non-classical cadherins. These included *fat* (*ft*) (Mahoney *et al.* 1991), *dachsous* (*ds*) (Clark *et al.* 1995), *Calsyntenin-1* (*cals*) (Vogt *et al.* 2001), and *Cad89D*, *Cad87A*, *Cad74A*, *Cad96Ca*, *Cad96Cb*, *Cad99C*, *Cad88C*, and *Cad86C* (Hynes and Zhao 2000; Tepass *et al.* 2000; Hill *et al.* 2001) (Supplementary Table S8 and Figure 2C). While the developmental roles for many of these non-classical cadherins have not been well studied, others have established roles in morphogenesis. Both Ft and Ds are regulators of planar cell polarity (Matakatsu and Blair 2004; Simon 2004) and are components of the Hippo signaling pathway (Hariharan and Bilder 2006; Willecke *et al.* 2006; Zhao *et al.* 2013), which is required for pupal eye patterning (DeAngelis *et al.* 2020). During larval eye development, *Cad86C* is a downstream target of Hh and Dpp signaling and is required for apical cell constriction during morphogenetic furrow progression, while both *Cad99C* and *Cad74A* are required for the organization of the follicular epithelium during development of the *Drosophila* oocyte (Schlichting and Dahmann 2008; Zartman *et al.* 2008; Chung and Andrew 2014). It could be that Cad86C, Cad99C, and Cad74A are required to maintain epithelial cell shape and integrity during pupal eye development as they do in the larval eye and oocyte. In particular, our results showed a large increase in the expression of *Cad89D* at 40 h relative to 21 h APF (log_2_-fold change = 9.36), suggestive of a prominent role for this cadherin during later stages of pupal eye development. Predictive bioinformatics analyses suggest that it binds to the members of the myosin family (Gaudet *et al.* 2011) and it will be interesting for future studies to identify its specific functions along with those of other non-classical cadherins in the developing pupal eye.

### Differential expression of cell survival genes

Programmed cell death is a critical aspect of pupal eye morphogenesis that occurs between ∼18 and 33 h APF and leads to the culling of roughly one in three lattice cells (Miller and Cagan 1998; Rusconi *et al.* 2000; Cordero *et al.* 2004; Lin *et al.* 2004; Mendes *et al.* 2006; Monserrate and Brachmann 2007; Verghese *et al.* 2012; Denton and Kumar 2015; Bushnell *et al.* 2018; DeAngelis *et al.* 2020). We therefore reasoned that we would observe decreased expression of established initiators or mediators of apoptosis at 40 h relative to 21 h APF and indeed this was observed for *klumpfuss* (*klu*) (Rusconi *et al.* 2004), *head involution defective* (*hid*) (Grether *et al.* 1995), *reaper* (*rpr*) (White *et al.* 1994), *Death Regulator Nedd2-like caspase* (*Dronc*) (Dorstyn *et al.* 1999), and *Death related ICE-like caspase* (*Drice*) (Fraser and Evan 1997) (Supplementary Table S9). Our analyses also identified a significant decrease in the expression of *Diap1* (Supplementary Table S9 and Figure 2C), an antagonist of apoptosis (Hay *et al.* 1995) that may be less critical at 40 h APF since apoptosis has abated and the transcription of known apoptotic inducing factors has decreased.

### Genes associated with muscle development and the sarcomere are required for pupal eye patterning

**Figure 4:**
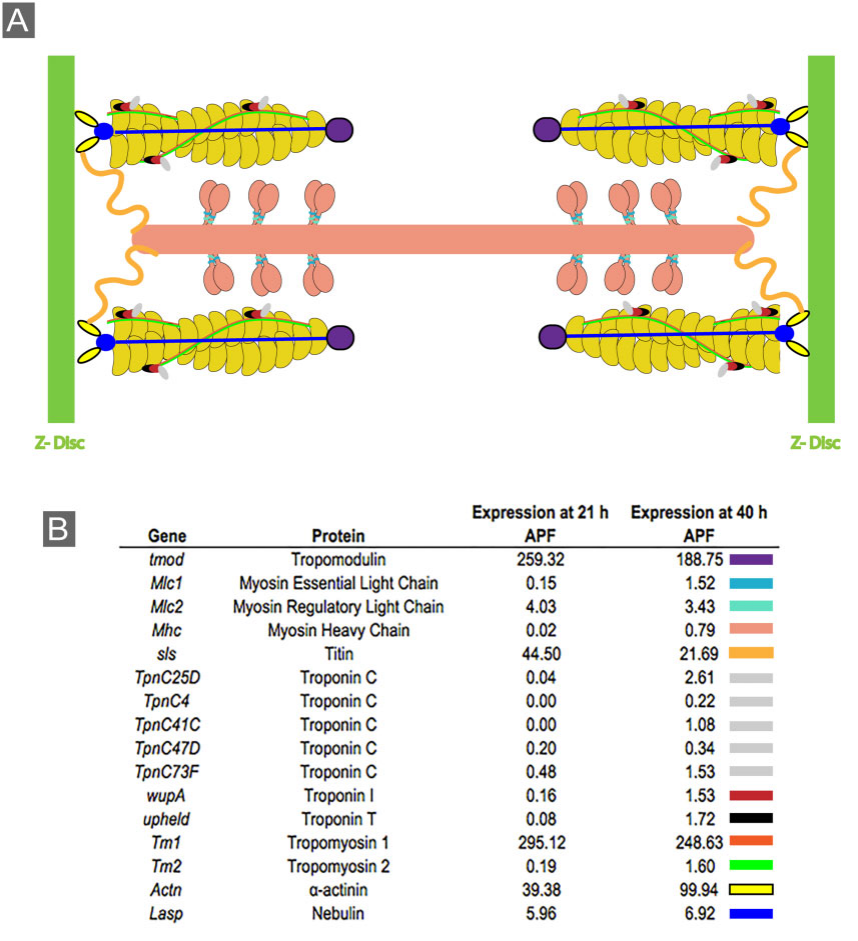
Genes associated with muscle function are expressed in the pupal eye. (A) Schematic representation of a sarcomere, composed of actin filaments (yellow), myosin bundles (peach) and a myriad of proteins that maintain sarcomere structure or function. (B) Relative expression (fragments per kilobase of transcript per million mapped reads) of sarcomere genes in the *Drosophila* eye at 21 and 40 h APF. Colored rectangles (at right) indicate color of protein in illustration.

Our RNA-seq analyses identified changes in the expression of many genes implicated in various aspects of muscle development or structure (Supplementary Table S10). These included *rst*, *hbs*, and *sns*, which are required for myoblast fusion during muscle development (Bour *et al.* 2000; Artero *et al.* 2001; Strünkelnberg *et al.* 2001), and *Klarsicht* (*Klar*), which is required for nuclei positioning in muscle cells (Elhanany-Tamir *et al.* 2012) and also has a documented role in photoreceptor morphogenesis (Mosley-Bishop *et al.* 1999; Patterson *et al.* 2004). Particularly, striking was the component of the muscle sarcomere, which facilitates muscle contraction, that were expressed in the developing pupal eye (Figure 4). Each sarcomere is composed of actin and myosin bundles along with regulatory proteins such as the troponins and tropomyosins, which mediate the interactions between actin and myosin bundles (Henderson *et al.* 2011; Sweeney and Hammers 2018; Mukund and Subramaniam 2019). Our analyses identified increased expression of core sarcomere components including *Myosin alkali light chain 1* (*Mlc1*) (Falkenthal *et al.* 1984), *wings up A* (*wupA*), which encodes the orthologue of Troponin I (Figure 2C) (Prado *et al.* 1995), and *Tropomyosin 2* (*Tm2*), which encodes a Tropomyosin that functions cooperatively with Troponin I during muscle contraction (Karlik and Fyrberg 1986; Naimi *et al.* 2001) (Figure 4). Since sarcomere-like structures are not found in the pupal eye, we predict that these proteins are repurposed for other roles in *Drosophila* retinal cells. For example, *wupA* and *Tm2* are required for the maintenance of chromosomal integrity and cell polarity in the syncytial embryo, S2 cells, and larval wing epithelia (Sahota *et al.* 2009; Casas-Tintó and Ferrús 2019). Previous studies also showed that *Mlc1*, *wupA*, and *Tm2* can interact in contexts other than the muscle sarcomere such as in S2R cells (Guruharsha *et al.* 2011). Further analyses are needed to clarify the precise molecular functions of these muscle-associated proteins during pupal eye morphogenesis.

### Computational identification of putative transcriptional regulatory factors

Our transcriptome analyses identified 4636 differentially expressed loci that contribute to multiple biological processes (Supplementary Tables S2–S4). To gain insight into common regulatory elements that might be utilized to facilitate changes in gene expression, we used full i-Cis target analysis (Herrmann *et al.* 2012) to identify transcriptional regulatory networks within groups of functionally related differentially expressed genes (documented in Supplementary Tables S5–S10). These analyses identified transcription factor-binding sites that were common to genes associated with signaling, axon guidance, biological adhesion, or muscle structure development, suggesting that these processes are regulated by a limited number of transcription factors (Supplementary Table S12). In contrast, we did not identify recurrent transcription factor regulatory motifs in the groups of genes associated with ecdysone signaling or cell survival that changed in expression from 21 to 40 h APF. This suggests that in the eye the genes associated with cell survival/apoptosis and ecdysone signaling are not a part of regulatory networks but instead their expression is regulated by multiple transcription factors.

The conserved GAGA transcription factor, *Trithorax-like* (*Trl*) (Farkas *et al.* 1994), emerged in our i-Cis analysis as a transcriptional regulator of loci associated with multiple processes including signaling, axon projection, adhesion, and muscle structure and development (Supplementary Table S12 and Figure 2C). A potential role for Trl was further suggested by using AME, which identified transcription factor binding motifs that were enriched in the complete list of differentially expressed genes. These analyses identified several transcription factors, including Trl, which may contribute to transcriptional regulation during pupal eye development (Supplementary Table S13) (McLeay and Bailey 2010). In a previous study of the role of Trl in the apoptosis of lattice cells, patterning defects are also evident in *Trl* mutant clones, underscoring a role for Trl in pupal eye morphogenesis (Dos-Santos *et al.* 2008). Trl can influence transcription by functioning as either a transcriptional activator (Biggin and Tjian 1988; Soeller *et al.* 1993; Farkas *et al.* 1994) or a repressor (Farkas *et al.* 1994; Horard *et al.* 2000; Busturia *et al.* 2001; Poux *et al.* 2001; Mahmoudi *et al.* 2003; Mishra *et al.* 2003), and interestingly, our analyses indicated that *Trl* expression declined significantly at 40 h relative to 21 h APF (Supplementary Tables S12 and S13). This change in *Trl* expression likely contributes to the very different transcriptional profile of the eye at 40 h APF (Supplementary Table S1).

In addition to Trl, our i-Cis analysis identified the transcription factor Homothorax (Hth) as a putative regulator of genes associated with axon guidance and muscle structure as well as Zelda (Zld), which may contribute to the expression of genes associated with axon guidance and adhesion (Supplementary Table S12 and Figure 2C). Frequently associated with the Hippo pathway (Peng *et al.* 2009), Hth is also required for the establishment of the eye field and photoreceptor specification (Pai *et al.* 1998; Pichaud and Casares 2000; Wernet *et al.* 2003; Singh *et al.* 2011). Hth has also been implicated in muscle fiber formation, which correlates with our association of Hth with the transcription of muscle-associated genes in the pupal eye (Bryantsev *et al.* 2012). The role of Zld during eye development has not been explicitly characterized during the pupal stage; however, previous studies indicate that it is expressed in the larval eye disk (Giannios and Tsitilou 2013). Another study reported that mutations in *zld* led to small and deformed eyes (Hamm *et al.* 2017). Like Trl, both Hth and Zld function as either transcriptional activators or repressors and both declined in expression at 40 h APF (Inbal *et al.* 2001; Kobayashi *et al.* 2003; Liang *et al.* 2008; McDaniel *et al.* 2019). Future studies will be needed to identify the transcriptional targets of Trl, Hth, and Zld during pupal eye morphogenesis.

To conclude, the pupal eye is an effective model system to use to interrogate processes required for the patterning or organization of epithelia as well as pathways that lead to cell differentiation, and photoreceptor morphogenesis and axon projection. However, while many of the morphological changes associated with these events have been well documented, less is known of the corresponding transcriptional changes that drive them. Here, we compare the transcriptomes of pupal eyes at two distinct stages of development. We identified changes in the expression of loci that are documented regulators of pupal eye development such as components of Notch and EGFR signaling pathways (Supplementary Table S5), ecdysone response targets (Supplementary Table S6), regulators of axon guidance (Supplementary Table S7), adhesion (Supplementary Table S8), and cell survival (Supplementary Table S9). In addition, we identified numerous novel gene expression changes that have not been studied in the context of pupal eye morphogenesis. These included components of the Toll pathway (Supplementary Table S5), non-classical cadherins (Supplementary Table S8), and numerous proteins required for muscle development and structure (Supplementary Table S10). We anticipate that these data will be a rich resource for future research on pupal eye morphogenesis and, given the highly conserved nature of many genes associated with tissue patterning, for the broader morphogenesis field as well.
